# AI Model for Detection of Abdominal Hemorrhage Lesions in Abdominal CT Images

**DOI:** 10.3390/bioengineering10040502

**Published:** 2023-04-21

**Authors:** Young-Jin Park, Hui-Sup Cho, Myoung-Nam Kim

**Affiliations:** 1 Division of Electronics and Information System, Daegu Gyeongbuk Institute of Science and Technology (DGIST), Daegu 42988, Republic of Korea; yjpark@dgist.ac.kr; 2 Department of Biomedical Engineering, School of Medicine, Kyungpook National University, Daegu 41566, Republic of Korea

**Keywords:** abdominal CT, abdominal hemorrhage, classification, detection lesion, deep learning

## Abstract

Information technology has been actively utilized in the field of imaging diagnosis using artificial intelligence (AI), which provides benefits to human health. Readings of abdominal hemorrhage lesions using AI can be utilized in situations where lesions cannot be read due to emergencies or the absence of specialists; however, there is a lack of related research due to the difficulty in collecting and acquiring images. In this study, we processed the abdominal computed tomography (CT) database provided by multiple hospitals for utilization in deep learning and detected abdominal hemorrhage lesions in real time using an AI model designed in a cascade structure using deep learning, a subfield of AI. The AI model was used a detection model to detect lesions distributed in various sizes with high accuracy, and a classification model that could screen out images without lesions was placed before the detection model to solve the problem of increasing false positives owing to the input of images without lesions in actual clinical cases. The developed method achieved 93.22% sensitivity and 99.60% specificity.

## 1. Introduction

AI is the art of creating computer systems that can perform tasks that utilize human intelligence and is already being used in many areas of society with a high impact. Machine learning (ML), a subfield of AI, uses computer science and statistics to learn from past experiences or data to improve future performance, and can automatically modify or improve algorithms to perform complex tasks without external assistance [1]. Deep learning (DL), a subfield of ML, has superior self-learning and self-debugging capabilities compared with traditional ML, enabling it to classify and predict with high accuracy, and is widely used in fields such as image processing and computer vision. Currently, convolutional neural networks (CNN), developed for improved feature extraction from large datasets, are widely used in deep learning [2,3].

After the ImageNet [4] project and the establishment of the structure of CNN, a neural network was designed based on the principle of how the human brain processes and recognizes images. Research on image classification and detection of specific objects in images has received considerable attention, and the number of studies has been increasing. In particular, the use of DL in medical imaging is growing rapidly because objects such as lesions and organs in medical images have complex geometries that are difficult to represent or detect accurately using simple equations or models [5]. Therefore, image diagnosis using AI improves the reliability of diagnosis and the utilization of medical resources according to the accuracy of lesion detection; therefore, there is increasing interest and support from the government, mainly from developed countries. Researchers are actively using AI technology to detect lesions such as cancer in organs such as the lungs and breasts in images such as CT and radiography or to detect brain aneurysms and brain tumors in magnetic resonance imaging (MRI) images, and highly accurate results are currently being reported. Radiological evaluation of a disease is primarily based on visual assessment, the interpretation of which can be augmented by advanced computerized analyses. AI is expected to assist specialists in qualitatively interpreting diseases, including delineating tumor volume over time, estimating biological progression, predicting clinical outcomes, and assessing the impact of disease and treatment on adjacent organs [6].

In this study, we focused on using AI to detect abdominal hemorrhage (AH) lesions with high urgency on abnormal CT images. Bleeding can manifest in different ways depending on the cause and anatomical location and is a common but serious medical emergency that requires early detection and appropriate intervention [7]. AH is defined as intra-abdominal bleeding and is often diagnosed based on radiologic findings, as the clinical symptoms are non-specific. In particular, due to its rapid and widespread availability, CT imaging plays an important role in analyzing and evaluating the presence, location, and extent of bleeding to determine the underlying cause [8]. In the field of imaging diagnosis, there is a need for methods and systems that can assist in the diagnosis of AH by quickly providing automatic analysis and reading information to medical staff in situations where no radiologists are present to read CT images or in emergency situations that may pose a serious threat to patients. Therefore, this study aimed to detect active bleeding foci and hematomas, which are formed by blood pooling after active bleeding, with high accuracy in abdominal CT images using DL technology.

The Abdominal CT Database (Abd-CT-DB) used in this study was collected from Seoul National University Hospital (SNUH) and Kyungpook National University Hospital (KNUH), both national hospitals in the Republic of Korea, where radiologists read each image, annotated the coordinates of the AH lesion, and provided them with the image. The CT images were saved in the Digital Imaging and Communications in Medicine (DICOM) standard format, and the annotations that stored the coordinates of the AH lesions were saved in the JavaScript Object Notation (JSON) format. Unlike natural images, medical images have different lesion shapes and patterns for each patient; therefore, it is necessary for radiologists to determine the location of the lesion by considering the specificity of the medical image. The method of detecting the location of AH lesions was implemented by feeding the dataset obtained by processing the Abd-CT-DB into a neural network for training. Abd-CT-DB converts the raw data in the DICOM file into a form that can be used in DL by visualizing the blood and soft tissue to be observed and generates a dataset by processing each image to map the location of the lesion. The dataset contains active bleeding and hematoma lesions of various sizes, not distinguished by type and size, and is randomly divided into training and test sets for the training and evaluation of the DL model.

The presence of small lesions in images can decrease the detection accuracy for the entire lesion. In this study, we designed a model that can detect AH lesions in abdominal CT images with high accuracy by improving the structure of the neural network to facilitate the detection of small lesions. In modern detection algorithms used for object detection, the neural network is fed only to images containing lesions, that is, images with correct answers, and is trained to find features and locations using regions predicted to be likely to contain lesions. However, in actual clinical practice, images containing lesions and images without lesions are utilized simultaneously; therefore, the detection model detects the location of lesions in all images, regardless of whether they contain lesions. In general, because the detection model is designed using a neural network to increase the number of true positives, false detection of lesions in images may increase when images without lesions are fed. To solve this problem, this study used an AI model with a cascade structure by assembling a classification model trained to classify the presence or absence of lesions and a detection model trained to detect lesions in images. In other words, for all input images, regardless of the presence or absence of lesions, the classification model is first used to classify the presence or absence of lesions before detecting lesions in each image; then, only the images determined to contain lesions are fed into the detection model to reduce the number of false positives.

In the evaluation, the accuracy of detecting the location of the lesion was determined based on the degree of overlap of the ground truth bounding box (GT-Box), which is the area detected by the radiologist, and the predicted bounding box (P-Box), which is the area detected by the implemented AI model according to the preset criteria. In the evaluation results of the AI model, the sensitivity, the probability of detecting the location of AH lesions, was approximately 0.93, and the specificity, the performance of screening images without lesions, was approximately 0.99. Therefore, the method proposed in this study can be used as a technical tool to provide meaningful diagnostic aid to medical staff in actual medical emergencies.

Since the abdominal CT images fed into the AI model do not know the presence or absence of AH lesions in clinical practice and the size distribution of lesions on images with lesions, the sensitivity and specificity used as performance metrics for the AI model may be reduced. In this study, we develop a detection model that detects AH lesions distributed in various sizes with high accuracy to increase sensitivity and develop an AI model with a cascade model structure to increase specificity to solve the problem of increasing the probability of false detection due to feeding in images without lesions.

The remainder of this paper is organized as follows. Section 2 introduces related work, and Section 3 describes the proposed method. Section 4 presents the evaluation results of the proposed method, followed by a discussion in Section 5. Finally, Section 6 concludes the paper.

## 2. Related Works

This section describes the research related to the proposed method to accomplish the challenging task of medical imaging diagnostics to detect AH lesions in CT images using DL.

The use of DL in medical image diagnosis is classified into the following: classification, which determines the presence or absence of a lesion in a medical image; detection, which detects the location of a lesion; and segmentation, which detects the shape of a lesion. The AI model developed in this study uses classification and detection together. The classification field is dominated by convolutional neural networks (CNNs) and derived networks [9]. An effective way to use CNNs for medical image classification is to use transfer learning to fine-tune CNN models pretrained on natural image datasets [10]. The study in [11], which showed the results before and after applying transfer learning to classify liver lesions in CT images, showed that, by applying transfer learning, the classification accuracy of AlexNet [12] improved from 78.23% to 82.94% and the classification accuracy of ResNet [13] improved from 83.67% to 91.22%. The results show that the application of transfer learning can improve the performance of the model, and the deepening of the network makes the image features extracted by the CNN model more detailed; thus, the classification accuracy of ResNet for medical images is considerably higher than that of AlexNet and ImageNet. There have also been studies on unsupervised learning for medical image enhancement using generative adversarial networks (GANs) [14] to classify lesions [15].

In the field of detection, RetinaNet [16], a one-stage detector used to solve the imbalance problem in which the background contains many regions containing objects, and Faster-RCNN [17], a two-stage detector that additionally utilizes a network to extract candidate regions, have shown excellent performance and are widely used in lesion detection research. A study [18] comparing the performance of RetinaNet with previous state-of-the-art one-stage detectors, Deconvolutional Single Shot Detector (DSSD) [19] and Faster R-CNN, showed results of 39.1 (RetinaNet) vs. 33.2 (DSSD) vs. 36.8 (Faster R-CNN), with RetinaNet achieving the highest mAP. In addition, many studies have combined multiple DL models, such as a study [20] that combined RetinaNet, an anchor-based detector, and VFNet [21] and FoveaBox [22] models, which are anchor-free detectors, achieving 91.69% sensitivity by detecting lesions in images provided by the DeepLesion [23] archive containing 32,000 CT images released by the NIH Clinical Center. A study on liver lesion detection using faster R-CNN based on an optimized squeeze net [24] achieved an AP of 63.81% on images provided by DeepLesion, showing a performance improvement of more than 20% over the baseline squeeze net.

In particular, the detection of small lesions that are difficult to detect and a reduction in false positives, which are incorrect detection results, are areas of active research in the field of image diagnosis. The abdominal hemorrhage dataset used in this study is a mixture of active bleeding and hematoma, which is a condition in which bleeding is in progress. The earlier the bleeding occurs, the smaller the size of the bleeding spot, which can be difficult to distinguish with the naked eye from other normal tissues around it. Therefore, detecting small lesions with active bleeding or hematomas on images has important clinical implications. In a study on small lesion detection in medical images [25], DeepLesion classified 32,735 bone, abdomen, mediastinum, liver, lung, kidney, soft tissue, and pelvic lesions across the body into small (less than 10 mm), medium (10–30 mm), and large (>30 mm) lesions. Subsequently, a deep learning model was constructed between ResNet-50 and the Feature Pyramid Network (FPN) [26] by changing the size and aspect ratio of the anchor and the network that applied the Attention Gate (AG) [27,28] to detect lesions of various sizes and structures to form the RetinaNet. Compared with 3DCE in a study [29] that demonstrated the state-of-the-art performance of DeepLesion, the sensitivity improved from 80% to 88.35% for small lesions, from 87% to 91.73% for medium lesions, and from 84% to 93.02% for large lesions.

The study in [30], which organized a pulmonary nodule detection system for false-positive reduction, consisted of a candidate detection phase to detect all suspicious pulmonary nodules and a processing phase to process the extracted patches for various candidate lesions using multiview ConvNets, showing a sensitivity of 90.1% on the Lung Image Database Consortium (LIDC-IDRI) [31] dataset. A study [32] on the problem of easily generating higher false positives while using an unbalanced dataset for lung nodule detection showed that introducing a filtering step to remove irrelevant images from the dataset can reduce false positives and increase the accuracy by more than 98%. The study in [33] proposed a 3D-CNN framework to extract more features by encoding richer spatial information compared with 2D to reduce false positives in automatic pulmonary nodule detection and obtained a sensitivity of 94.4% in the false-positive reduction track of the LUNA 16 Challenge using the LIDC dataset.

Hemorrhage detection has been studied in the fields of intracerebral hemorrhage and retinal fundus. A study on the detection of intracerebral hemorrhage [34] used deep learning segmentation for more accurate detection of the hematoma volume. In the field of retinal fundus using the DIARETDB1 database, a public database used for diabetic retinopathy detection, research [35] presented an automatic hemorrhage detection method based on two-dimensional Gaussian fitting and obtained 100% sensitivity, 82% specificity, and 95.42% accuracy using 219 retinal fundus images. The study in [36] used a scale-based method to segment blood vessels from hemorrhages and finally used gamma correction and thresholding methods to detect hemorrhages, achieving 87% sensitivity, 84% specificity, and 89% accuracy. However, most studies on the abdomen aim to localize and segment organs, mainly the liver, kidneys, bladder, and pancreas, whereas MRI is used for prostate analysis, and CT is predominantly used for all other organs [37].

Because previous studies on AH lesion detection using DL are currently difficult to find, it was not possible to directly compare and evaluate their contributions, and it was found that there may be various problems related to lesion detection using DL; for example, the detection performance may vary depending on the size of the lesion or the problem caused by an increase in false positives. Therefore, we propose a method to solve the problems presented in the following section.

## 3. Methods

In this study, we processed Abd-CT-DB data to generate a dataset consisting of data suitable for use in DL, developed a neural network for a detection model that detects AH lesions of various sizes with high accuracy, and developed an AI model with a structure that places a classification model before detection to reduce false positives.

### 3.1. Creation of the Dataset

**Figure 1:**
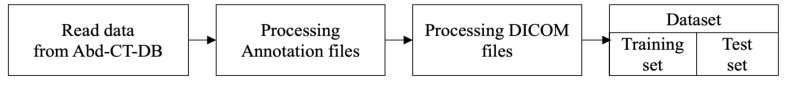
The processing flow of annotation and DICOM files for creating the dataset for use in DL.

The processing of the Abd-CT-DB involves cleaning and structuring data from annotation files and raw DICOM files with errors in meta-information or problems in representing the geometry of lesions to create a dataset, as illustrated in Figure 1.

#### 3.1.1. Processing of Annotation and CT Images

Because there were cases in which there were problems with the information in the annotation file, such as errors in the coordinate information provided or the absence of a corresponding DICOM file, the coordinates of the lesion location were extracted only for images with accurate information to avoid a mismatch between the annotation and DICOM files. The images with extracted coordinates were divided into abnormal datasets consisting of abnormal slices with lesions and normal datasets consisting of normal slices without lesions; the coordinate extraction process was omitted because the annotation file was not provided for the normal dataset.

For the DICOM files, we utilized the header attribute information of each image to apply the rescaling method to place the luminance values in the same region and the windowing method to extract only the luminance values within the region of interest, except for images with errors, for images with widths and heights of 512 × 512 pixels. The Hounsfield unit (HU) [38] represents the degree of attenuation in the transmission of X-rays for a given image and is universally used in CT scans to represent CT figures in a standardized and convenient form [39]. CT images are expressed relative to other areas, with the attenuation of water being zero, and each pixel typically has a luminance value in the range of 12 bits (4096 levels). Rescaling is the process of placing images in the same luminance region using Equation (1), where the rescale slope represents the slope of the pixel values for each image, and the rescale intercept represents the offset and includes a negative sign. For example, an image with a luminance range of 0 to 4095 would be rescaled to a range from −2048 to 2047 because the rescale slope is set to 1 and the rescale intercept is set to −1024. However, the image in the range from −2048 to 2047 is unchanged because the rescale intercept is 0, so the two images have the same luminance region.

(1) real image=rescale slope × saved image+rescale intercept

Windowing is a method used to highlight specific tissues in an image, such as X-ray or CT images, by setting a range of tissues in the region of interest for observation using the window center and width properties, which are expressed differently depending on the setting. In the images used in the study, soft tissue and blood regions are represented as the regions of interest; in the dataset used, the window center has a value between 30 and 80, and the window width, which represents the HU range of the region of interest, has a value between 400 and 450.

**Figure 2:**
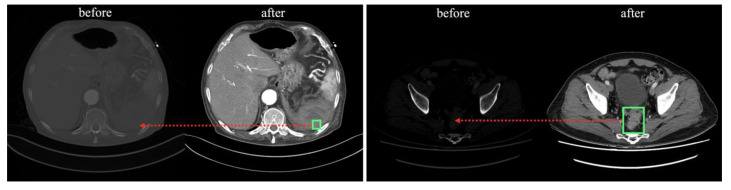
Comparison of before and after processing of DICOM file.

The processed images were normalized so that each pixel was linearly stretched by fitting a value between 0 and 1, and stored as a 512 × 512 pixel NumPy format array. The location of the lesion (green box) and structures, such as various organs in the abdomen are more visually distinct (red arrows) compared with the pre-processed images in Figure 2.

#### 3.1.2. Materials

The Abd-CT-DB contains data from 515 patients (SNUH:360, KNUH:155) and was approved by the Institutional Review Board (IRB). Table 1 shows the dataset constructed by processing Abd-CT-DB. The datasets of the two hospitals were merged and used for DL training and evaluation. All coordinate information extracted from JSON was randomly shuffled and saved as a single annotation file, which was divided into a training set and a test set at a ratio of 9:1, along with the corresponding NumPy files, and stored on a disk.

Table 2 shows the distribution of lesions in each dataset by size. All lesions were <160 × 160 pixels in size. In this study, lesions with a size of 40 × 40 pixels or less were defined as small lesions. The SNUH dataset was mostly composed of small lesions, whereas the KNUH dataset had the opposite composition. The merged dataset had an appropriate mix and distribution of lesion sizes. Therefore, it was organized to detect lesions of all sizes without distinguishing between them.

### 3.2. Development of the AI Model Using Deep Learning

**Figure 3:**
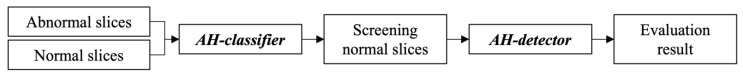
Flow of AH lesion detection using AI models.

The AI model is designed as a cascade model structure with an AH classifier, a classification model, and an AH detector, a detection model that detects the location of the lesion by feeding the merged dataset in Table 1 to classify the presence or absence of the lesion in advance, as shown in Figure 3. After describing how to develop a detection model for AH lesions, this section describes the deployment of the model for lesion classification.

#### 3.2.1. The AH Detector to Improve Small Lesion Detection Performance

To improve the detection accuracy for small lesions, the researchers developed a neural network and adjusted the parameters of the anchor box to fit the dataset.

**Figure 4:**
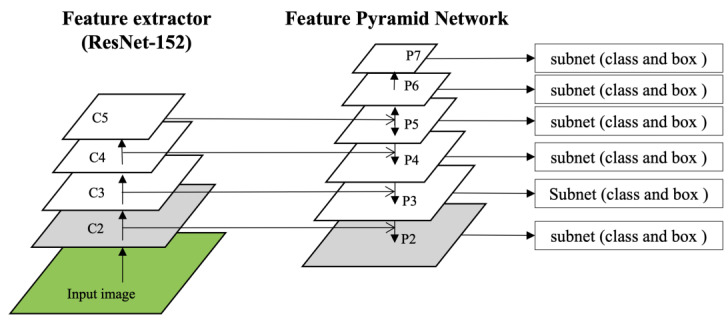
The structure of the improved neural network for small lesion detection.

The neural network designed to recognize the lesion patterns in the detection model was composed of ResNet-152 with a bottom-up pathway and an FPN with a top-down pathway, as shown in Figure 4. The FPN is separated by the layer specified by the CNN to generate a feature map, and the closer it is to the input layer, the higher the resolution that retains the feature information, such as edges and curves of the image, and the farther it is from the input layer, the less it loses the features that can be inferred through texture or parts of the object. The detection of small objects using DL is a challenging task in computer vision, which requires sufficiently high-resolution images to recognize small objects [40]. The neural network developed in this study adds a C2 layer to the convolutional network and a P2 layer to the pyramid network to increase the resolution of small lesions by changing the number of utilized layers to four levels between C2 and C5, and six levels between P2 and P7. For reference, the baseline RetinaNet consisted of three levels between C3 and C5 and five levels between P3 and P7.

An anchor box is used in detectors such as RetinaNet to detect the location of objects during training, using predefined areas, aspect ratios, and scale parameters for each feature map, and the GT-Box and Intersection of Union (IoU) [41] to classify lesions and backgrounds as positive and negative anchors, respectively. Positive anchors are utilized for both classification and box regression training to predict both the detected coordinates and class for a specific lesion, whereas negative anchors are utilized only for classification because they do not contain information about the lesion but only about the background. The two subnetworks that perform classification and bounding box regression have the same structure but use different parameters; therefore, they do not share information with each other. The anchor area is the area of the anchor box in each feature map, and the aspect ratios define the shape of the anchor box. The scales were calculated from the areas of each feature map and used to create anchor boxes of different sizes, which should be used according to the size of the lesion, as higher resolution detects smaller lesions using smaller anchor boxes. The baseline RetinaNet anchors used the default areas {32, 64, 128, 256, 512}, aspect ratios {1:2, 1:1, and 2:1}, and scales {1, 1.26, 1.59} for P3 to P7. However, if the parameters are not properly tuned to the size of the lesions to be detected, the bias may be detrimental to learning and may need to be adjusted to match the characteristics of the training dataset, such as a large number of small lesions or, conversely, a large number of large lesions in a particular dataset [42].

Because the dataset used in our study had lesions of different sizes, we adjusted the parameters to improve the detection accuracy for smaller lesions. The area of each feature map is {16, 32, 64, 128}, corresponding to P2–P5. The aspect ratios were obtained by distributing the ratios of all lesions in the dataset to each area range of 20 × 20 or less and 40 × 40 or less, and obtaining the area range of the lesion with the most distributed ratios, {1, 1.81, 2.75}, and {2.75:1, 1.81:1, 1, 1:1.81, 1:2.75}. For the scales, we divided the area of all lesions in the dataset by the area of each feature map and used {0.4, 0.55, 0.70} between [0.2, 1.3], which is the range of the mean values of the distribution for the extent to which an anchor box can cover a lesion in each feature map.

#### 3.2.2. Placement of the AH Classifier to Reduce False Positives in AI Models

**Figure 5:**
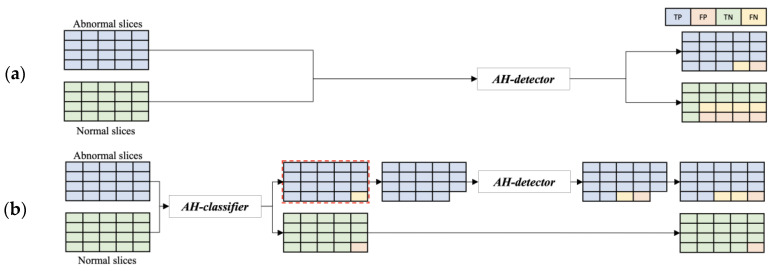
Example comparison of single and cascade model with and without AH classifier placement: (**a**) single model; (**b**) cascade model.

Detection models are typically designed to detect images with lesions; however, in real-world clinical practice, images with lesions and images without lesions may be fed in, thereby increasing the number of false positives. Figure 5 illustrates an example of the single-model method with the configuration shown in Figure 5a, where the dataset is directly fed into the developed AH detector, and the cascade model method with the configuration shown in Figure 5b, where the AH classifier designed with the CNN architecture with the ResNet-152 structure is deployed before detection. The structure of the cascade model method reduces the number of false positives compared with the single-model method, and the results of both models are used together to maintain the number of images initially fed. Therefore, this study solved the problem of increasing false-positive results by pre-positioning the AH classifier for screening normal images. The experimental process and results using the real dataset are described in the next section.

## 4. Experiment and Results

This section describes the training method of the AI model, numerical results of calculating the detection accuracy, and visualization results of outputting the GT-Box and P-Box on the actual detected images. The training and evaluation were performed in an environment consisting of the hardware and software listed in Table 3.

### 4.1. Training of Models in AI Model

**Figure 6:**
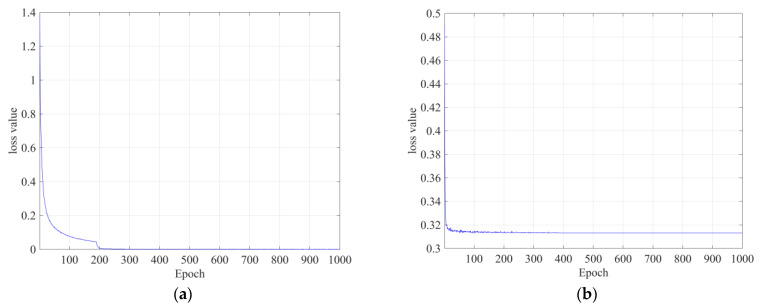
Training loss in each epoch: (**a**) detection models; (**b**) classification models.

The detection and classification models were trained using the same training parameters by setting the following parameters: size, 4; optimizer, Adam; and learning rate, 1 × 10^−5^. Transfer learning was used to overcome the lack of data. The detection model was developed by applying the improvement method to the baseline RetinaNet [43], where the lesion detection accuracy was not improved. The classification model using CNN was trained for 1000 epochs, and no problems such as overfitting occurred during the learning process, as shown in Figure 6a,b. The detection model uses a supervised learning method that uses the annotation results of radiologists as correct answers, whereas the classification model uses abnormal and normal classes as correct answers.

**Figure 7:**
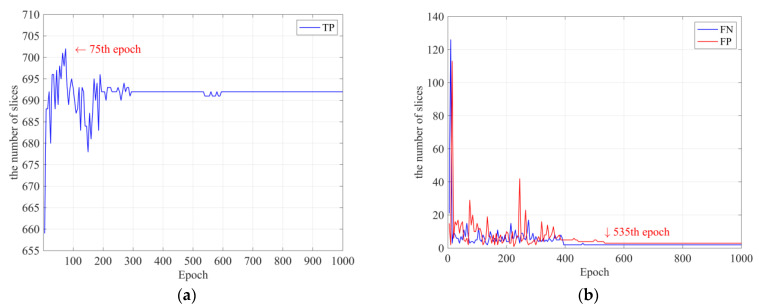
Training result of detection models and classification models created in each epoch: (**a**) the number of true-positive slice groups in detection models; (**b**) the number of false-positive and false-negative slice groups in classification models.

After training each model, the best-performing model was selected from the final confusion matrix consisting of true-positive (TP), false-positive (FP), true-negative (TN), and false-negative (FN) slice groups and was used as the lesion detection model AH detector and classification model AH classifier. The AH detector is the model generated at the 75th epoch (red arrow) in Figure 7a, which contains the highest number of TPs, and the AH classifier is the model generated at the 535th epoch (red arrow) in Figure 7b, which contains the lowest number of FPs and FNs.

### 4.2. Evluation Method

The detection of lesions using the trained model was evaluated using the confidence and IoU scores to determine performance. The confidence score indicates the probability of the presence of a lesion, which is used to first remove the overlap of many bounding boxes created around the location of the lesion and determines the use of the region based on whether a threshold is met. The IoU is a numerical representation of the intersection of the GT-Box annotated by the radiologist and the P-Box predicted by the model and is calculated by dividing the intersection of the two boxes by the sum of the two boxes, as shown in Equation (2).

(2) $$\mathrm{IoU}=\frac{\mathrm{area}\;\mathrm{of}\;\mathrm{intersection}}{\mathrm{area}\;\mathrm{of}\;\mathrm{union}}$$

Table 4 shows a representation of the detection conditions and confusion matrix for images with lesions fed into the AH detector. TP_D is a group of slices in which a lesion is detected because the confidence and IoU scores are greater than the threshold, FP_D is a group of slices in which the confidence score is greater than the threshold but the IoU score is less than the threshold, and FN_D is a group of slices in which the confidence score is less than the threshold and the lesion is incorrectly judged as a normal image. However, the TN used for normal images without lesions could not be represented in the confusion matrix of the AH detector. In this study, the threshold value of the confidence score was greater than 0.5 and the IoU threshold value was greater than or equal to 0.4.

Table 5 shows the confusion matrix for the predicted result (Predict) based on images with and without lesions (Target) fed into the AH classifier.

### 4.3. Evaluation Result

**Figure 8:**
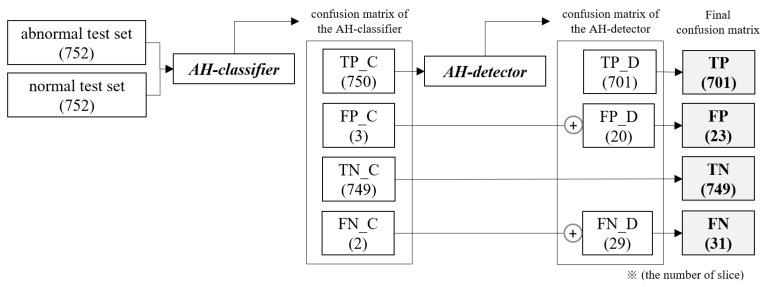
Confusion matrix generated for each step of the AI model for detecting AH lesions using the dataset.

Because the trained AI model has a cascade structure consisting of an AH classifier and an AH detector, the step-by-step flow of inputs and outputs according to the placement of each model, as well as the change in the number of slice groups derived as a result, are shown in Figure 8.

The AH classifier was fed the abnormal and normal test sets together to classify the presence or absence of lesions before detection, and the TP_C slice group classified as having lesions was fed into the AH detector to detect lesions. In the final confusion matrix representing the final result, TP is the number of TP_D slice groups in which lesions were detected by the AH detector, and TN is the number of TN_C slice groups classified as normal images by the AH classifier, indicating the classification performance for normal slices. FP and FN are the results of the two models added to maintain the number of slices in the initial test set without missing slices.

Sensitivity (true-positive rate, TPR) is the proportion of true positives correctly identified by the test, and specificity (true-negative rate, TNR) is the proportion of true negatives correctly identified by the test [44,45]. Sensitivity, which indicates the probability of abnormal slices with lesions, was calculated using Equation (3), and specificity, which indicates the probability of normal slices without lesions, was calculated using Equation (4).

(3) $$\mathrm{TPR}=\frac{\mathrm{number}\;\mathrm{of}\;\mathrm{the}\;\mathrm{true}\;\mathrm{positives}}{\mathrm{total}\;\mathrm{number}\;\mathrm{of}\;\mathrm{abnormal}\;\mathrm{slice}\;\mathrm{that}\;\mathrm{has}\;\mathrm{the}\;\mathrm{lesion}}\times 100\%$$

(4) $$\mathrm{TNR}=\frac{\mathrm{number}\;\mathrm{of}\;\mathrm{the}\;\mathrm{true}\;\mathrm{negatives}}{\mathrm{total}\;\mathrm{number}\;\mathrm{of}\;\mathrm{normal}\;\mathrm{slice}\;\mathrm{that}\;\mathrm{has}\;\mathrm{no}\;\mathrm{the}\;\mathrm{lesion}}\times 100\%$$

The evaluation results for the AI model are shown in Table 5 and Table 6, which compare the improvement in detection accuracy and reduction in false positives. The TPR of the AH detector for images with lesions improved compared with that of the baseline model, as shown in Table 6.

Table 7 compares the results of the AI model and AH detector for images with and without lesions and shows that the AI model with the AH classifier placed before detection improved the specificity and reduced the number of FPs compared with the AH detector. In the evaluation results of the AI model, the sensitivity, the probability of detecting the location of AH lesions, was 93.22%, and the specificity, the performance of screening normal images, was 99.60%. Therefore, the AI model developed in the study showed a statistically significant increase in both sensitivity and specificity in the experimental results for images with and without AH lesions, suggesting that it can be used in clinical applications to increase the probability of detecting AH lesions.

**Figure 9:**
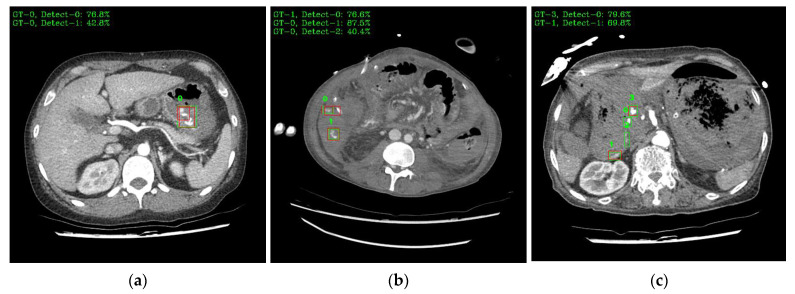

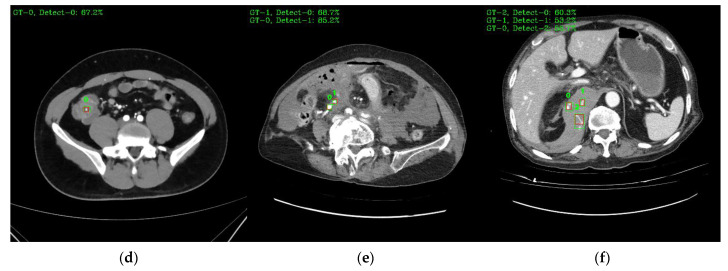
Example images of the AH-lesion detected using the AH detector. Green boxes indicate GT-Box, and red boxes indicate P-Box. Green text is GT-Box index, P-Box index, and IoU score: (**a**) single GT-Box and detected two P-Box; (**b**) two GT-Box and detected each single and two P-Box; (**c**) four GT-Box: detected two P-Box and missed two P-Box; (**d**) single GT-Box: detected single P-Box; (**e**) two GT-Box: detected two P-Box; (**f**) three GT-Box: detected three P-Box.

Figure 9 shows a visual representation of the results of lesion detection using the AH detector. Each image used for detection contains at least one GT-Box (green rectangle), which is the correct region. A P-Box (red rectangle), which is the detected region, is displayed according to the number of lesions detected and is not displayed when detection fails. The green text in the upper left corner of each image shows the index number of the GT-Box, index number of the detected P-box, and information about the IoU score. For example, Figure 9a shows that, for one GT-Box with a GT-Box index number of 0 (GT-0), two P-Box index numbers (Detect–{0, 1}) were generated with IoU scores of 76.8% and 42.8%. In addition, as shown in Figure 9c, out of the four GT-boxes with GT box index numbers GT–{0, 1, 2, 3}, only GT–{1, 3} was successfully detected, resulting in P-boxes with IoU scores of 69.8% and 79.6%, respectively.

## 5. Discussion

DL models have a high probability of achieving good results when sufficient quality data are available. However, most medical data are restricted by national or hospital policies regarding the utilization of patient information. Currently, research on the detection of AH lesions using AI technology is difficult because of the lack of effective related studies.

The main contributions of this paper are summarized as follows: The AI model developed in this study improves both sensitivity and specificity by designing a detection model to detect AH lesions distributed in various sizes with high accuracy and by deploying a classification model that can screen out images without lesions before detection to solve the problem of increasing the probability of false detection due to the feeding of images without lesions. In this study, we conducted an experiment to determine the effects of the methods applied to the development of an AI model on the increase in sensitivity and specificity. First, the AH detector achieved a sensitivity of 93.22% by changing the anchor parameters in the baseline model and by changing the number of utilized layers in the neural network to improve the detection accuracy for small-sized lesions. Therefore, the applied method was not significantly affected by the distribution of small lesions and could detect lesions in images with high accuracy. However, because the AH detector is designed to detect lesions in images with lesions, the number of false positives may increase in clinical cases where normal images are fed. The AI model, which is a cascade model structure, obtained an FP of 23 and a specificity of 96.60%, reducing the FP by 76 and increasing the specificity by 12.76% compared with a single model using only an AH detector. Therefore, the method of screening images without lesions to solve the problem of increasing the probability of false positives reduces the number of false positives for the input of images without lesions effectively.

The IoU is an important numerical value that quantifies the degree of overlap between the GT-Box and P-Box during the detection and evaluation of anchor boxes when training a detection model, and the relationship between the IoU score and threshold is an important factor in performance evaluation because the smaller the set IoU threshold value, the more P-boxes are removed. The IoU threshold value is usually 0.5 for both training and evaluation to determine detection. However, in this study, the threshold was set to 0.4 for evaluation to obtain more detection results, and the mid-stage experiment performed with the threshold set to 0.5, in the same environment, still showed a high performance with a sensitivity of 91.60%. The images in Figure 9, which show the results of visual reading, seem to be at a sufficient level to generate information, such as a critical value report (CVR), to assist in reading, even when the IoU score is detected at a value below 40%. Unless the area that detected the lesion is a completely different spot, it is more useful for the assistant system to show as many detections as possible to aid in the reading. Imaging diagnostics must be viewed from the perspective of the radiologist who actually operates and reads the images, and not just from the perspective of the engineer who focuses on the numbers. Medical decisions made by radiologists can be life-or-death decisions for patients; therefore, it is important to provide patients with sufficient cues when in doubt. Therefore, the IoU threshold is a matter for medical decision makers to set based on their own contextual judgments.

The utility of AI technology in clinical practice may require many resources to prove. Therefore, even if it is available after a long validation process, it is still expected to be used as a tool for redundant judgment. Imaging diagnosis using AI can only achieve good results with the active participation of radiologists with a high ability to read lesions, and AI models trained with data biased by the size and shape of lesions cannot perform well. Therefore, because the dataset used in this study did not represent all possible sizes and shapes of AH lesions, it is necessary to build a database that detects lesions of various sizes and shapes with the participation of as many radiologists as possible before it can be used in clinical practice.

## 6. Conclusions

DL technology, which is primarily used in image processing, is used to extract meaningful features from input images and to use them in neural networks. In the field of image diagnosis using DL technology, the question of liability can be an important issue in the event of a medical accident, so it is still mostly used as an aid to diagnosis. However, owing to the utility of rapid and accurate detection in emergency situations, image diagnosis methods using DL and their performance are required. In particular, there is a need for a system that automatically analyzes medical images when there are no radiologists to read the images or in emergency situations and that quickly provides readings to the medical staff to assist in diagnosis.

In this study, we developed an AI model to detect abdominal hemorrhagic lesions using CT. The AI model consisted of two models to detect AH lesions, and IRB-approved datasets from multiple national general hospitals in Korea were used for training and evaluation. The presence and size distribution of lesions is one of the most important factors in image analysis. In clinical applications where images are used that do not take into account the presence of lesions and their size distribution, AI models may have difficulty accurately recognizing lesions, resulting in a decrease in sensitivity and specificity due to an increase in the probability of not detecting lesions or false positives. In this study, a detection model was first developed to detect small lesions with high accuracy to increase sensitivity and an AI model with a cascade structure was designed to reduce false positives when inputting images without lesions. The developed AI model was designed to improve detection performance by changing the number of layers of the neural network in the detection model and anchor parameters optimized for the dataset used in the study and to reduce false positives by deploying a classification model to screen images without lesions before detection. The detection model increased sensitivity to AH lesions distributed in various sizes compared with the baseline model, and the AI model with a cascade structure increased specificity and reduced false positives compared with the single model structure.

Therefore, the developed method effectively detected abdominal bleeding of various lesion sizes with a sensitivity of 93.22%, which is the probability of detecting the location of the lesion, and a specificity of 99.60%, which is the performance of selecting images without lesions. However, a direct performance comparison could not be made because we could not find relevant studies on detecting the same type of lesions using AI technology. As a result, this study is expected to help researchers in the field of imaging diagnosis using AI technology to detect AH lesions, which lack relevant studies.

In future research, we will study medical image processing methods to improve accuracy by extracting feature points or preserving boundaries of lesions in images to improve detection accuracy for small lesions and to research how to improve deep learning algorithms.

## Figures and Tables

**Table 1:** The final dataset composed after processing of Abd-CT-DB.

Dataset	Abnormal Slices			Normal Slices		
	Train	Test	Total (%)	Train	Test	Total (%)
**Merged (Used)**	**6762**	**752**	**7514 (100)**	**6762**	**752**	**7514 (100)**
SNUH	3800	415	4215 (56.1)	3800	415	4215 (56.1)
KNUH	2962	337	3299 (43.9)	2962	337	3299 (43.9)

**Table 2:** Distribution of lesion sizes in a dataset.

Dataset	Lesion Size (≤)					
	20 × 20	30 × 30	40 × 40	60 × 60	80 × 80	160 × 160
**Merged (%)**	**2230 (21.58)**	**4164 (40.28)**	**5633 (54.49)**	**7829 (75.73)**	**8975 (86.82)**	**10,338 (100)**
SNUH (%)	2213 (42.86)	3926 (76.04)	4721 (91.44)	5114 (99.05)	5150 (99.75)	5163 (100)
KNUH (%)	17 (0.33)	238 (4.60)	912 (17.62)	2715 (52.46)	3825 (73.91)	5175 (100)

**Table 3:** The experimental environments of hardware and software for deep learning.

Hardware		Software	
CPU	IBM Power 9 3.8 GHz 16 cores 2 ways	OS	Ubuntu 18.04LTS
GPU	nVidia Tesla V100 3t2 GiB	CUDA (driver)	10.2 (440.64)
RAM	631GiB	Python	3.7.7
DISK	SSD 4TB	PyTorch	1.3.1

**Table 4:** The confusion matrix for the AH detector.

Slice Group	Detection	Condition
TP (TP_D)	Success	Confidence score > 0.5 and IoU score >= 0.4
FP (FP_D)	Fail	The number of total slices—TP—FN
FN (FN_D)	Fail	Confidence score <= 0.5

**Table 5:** The confusion matrix for the AH classifier.

Slice Group	Target	Predict	Classification Result
TP (TP_C)	abnormal	abnormal	correct
FP (FP_C)	normal	abnormal	incorrect
TN (TN_C)	normal	normal	correct
FN (FN_C)	abnormal	normal	incorrect

**Table 6:** Comparison between detection models to observe an increase in sensitivity and TP.

Model	TP	FP	FN	Total	TPR (%)
Baseline	550	9	193	752	73.10
AH detector	702	20	30	752	93.40

**Table 7:** Comparison between a single model and a model with a cascade structure to observe an increase in specificity and a decrease in FP.

Model	TP	FP	TN	FN	Total	TPR (%)	TNR (%)
AH detector	702	119	653	30	1504	93.40	86.84
AI model (cascade)	701	23	749	31	1504	93.22	99.60

## Data Availability

Not applicable.
